# Supplementary material for: Are carbon emissions trading and green financial instruments synergistic? -Comprehensive quantitative research based on content analysis
Source: PLoS One. 2024 Mar 7;19(3):e0298601. doi: 10.1371/journal.pone.0298601 (PMC10919601; doi:10.1371/journal.pone.0298601)
Supplement: S1 Data — (ZIP) [file pone.0298601.s001.zip › Data/地级市Z_F工作报告.zip!/╕ú╜¿/╧├├┼╩╨/└┤╫╘╣1⁄2╓┌║┼í╛┬φ┐╦╩2╛▌═°í┐_2015.docx]

各位代表:

　　现在，我代表市人民政府向大会报告工作，请予审议，并请各位政协委员和其他列席人员提出意见。

　　一、2014年工作回顾

　　过去的一年，我们在市委正确领导下，全面贯彻党的十八大和十八届三中、四中全会精神，全力稳增长、调结构、促改革、惠民生，加快实施美丽厦门战略规划，较好地完成全年各项任务。地区生产总值3273亿元，增长9.2%，增幅位居副省级城市前列；全社会固定资产投资1573亿元，增长16.7%，其中重点项目投资1025亿元；财政总收入909亿元, 增长10.2%，其中，地方级财政收入543亿元，增长10.8%；城镇居民和农民人均可支配收入分别为39625元和16220元，增长8.2%和10.6%；居民消费价格上涨2.2%；万元生产总值耗电、耗水分别下降1.4%和2.2%；年度节能减排任务可以完成。

　　一年来的主要工作和成效:

　　（一）转型升级步入新阶段

　　稳增长措施有力有效。面对复杂严峻的经济形势，我们坚持稳中求进，科学研判、多措并举，加大经济运行调度，及时出台稳定工业、扶持外贸、促进消费、鼓励民企等政策，实施支持软件信息服务、会议展览、服务外包发展等措施。挂钩服务大型工业、内外贸企业，帮助解决融资、用地、用工等问题，促进增资扩产。有针对性的措施拉动了增速逐月企稳回升，在促进经济平稳健康发展的同时，也为转型升级创造了有利条件。

　　调结构步伐加快。三次产业结构由上年的0.8:46.7:52.5调整为0.7:45.8:53.5。先进制造业集聚效应不断增强，全年工业总产值突破5000亿元，规模以上工业增加值1240亿元，增长10.5%，对经济增长贡献率达49.4%。机械、电子两大支柱产业产值3202亿元，占规模以上工业总产值65.3%。

　　平板显示产业链产值突破千亿，增长10%。软件信息业务收入增长24.3%，生物与新医药产业链产值增长11.3%，文化创意产业增加值增长12%。现代服务业发展提速、比重提高、水平提升，第三产业增加值1750亿元，增长8.7%，获批国家现代服务业综合试点城市。举办展览200场、会议4177场，均比往年有较大增长；接待境内外游客5338万人次，增长14.5%；旅游总收入722亿元，增长16.3%。规模以上物流企业营业收入增长38.7%，空港旅客吞吐量突破2000万人次，已开通国内航线182条、国际航线30条；海港货物吞吐量达2亿吨，集装箱吞吐量达857万标箱，分别增长7.4%和7.1%。金融机构本外币存、贷款余额分别为7065亿元和6644亿元，增长10.7%和13.7%，存贷比为94%。

　　创新驱动更加强劲。全社会研发投入占地区生产总值比重提高到2.9%。获国家各类科技立项508项，当年到位资助资金4亿元。新认定高新技术企业151家，省、市级创新型试点企业92家。规模以上高新技术工业企业产值 1984亿元，占规模以上工业总产值40.4%。稀土材料研究所和产业化基地正式立项。厦门获评全国半导体照明两家A类基地之一，生物医药、海洋高技术成为国家产业发展试点。全市各类专利授权8944件，每万人拥有有效发明专利11.1件。国家和省级重点实验室、工程技术研究中心、企业技术中心、博士后工作站累计达121家。主导或参与制定国家标准、行业标准130项。加大各层次人才引进，900多人纳入“海纳百川”人才计划。

　　智慧城市建设稳步推进。深入创建国家信息消费示范城市、信息惠民国家试点城市，获批国家数字家庭应用示范产业基地，入选宽带中国示范城市。三网融合试点工作取得成效，固定宽带接入超过150万户。软件园三期入驻企业357家。智慧社区提供社会救助、家庭安防、劳动就业等公共服务，智慧交通带来更便捷的出行服务，智慧教育实现宽带网络校校通，智慧医疗建成全市门诊预约统一平台和健康医疗云平台，智慧社保走在全国前列。

　　（二）改革迈出新步伐

　　行政领域改革力度加大。完成新一轮机构改革，政府工作机构由45个减至39个。理顺鼓浪屿管理体制，促进了整治提升和申遗工作。优化市对区财政管理体制，调动区级发展积极性。在教育、医疗等8个领域开展政府购买服务。精简、下放审批服务事项295项，市级保留329项，网上审批覆盖面进一步扩大。“多规合一”使建设项目审批效率大幅提高。

　　经济领域改革持续深化。商事登记制度改革起步早、力度大，实现证照分离，激发市场活力，新增商事主体7万多户，增长80%。民间投资增长41.5%，占全社会投资比重超过四成。制定外商投资审批清单，进一步放宽领域、简化手续。推动国企发展混合经济，利润总额增长13%。中航工业重组海翼集团，促进厦工产品转型升级。营改增试点范围扩大到铁路运输、邮政业和电信业。创新区域合作方式，与龙岩、安溪共建协作经济区。

　　社会领域改革不断拓展。设立科技成果转化与产业化基金，试行科技保险业务，科技投入向贷款贴息、担保贷款、股权投入转变。实施进城务工人员随迁子女积分入学，取消招收高中择校生。放宽医师多点执业，启动分级诊疗制度改革。扩大社会组织直接登记范围。开展农村土地承包经营权确权登记颁证工作，完善农村宅基地政策。

　　（三）开放型经济再上新台阶

　　利用外资获得重大突破。我市利用外资在国际经济形势低迷中逆势增长，全年新批外资项目416个，其中总投资超亿美元项目19个。总投资62亿美元的台湾联电12寸晶圆片项目获批落地，将推动集成电路下游产业集聚厦门。总投资7亿美元的日本电气硝子高世代液晶玻璃基板项目开工建设，使厦门平板显示产业集群更加完整。总投资120亿元人民币的天马微电子二期，将与一期项目配套形成产业优势。总投资20亿元人民币的ABB工业中心，是该集团在海外投资建厂的第一个项目。全年合同利用外资28.5亿美元，增长49.5%；实际利用外资19.7亿美元，增长5.3%，总量均居全省首位。

　　自贸试验区创建取得重大进展。国务院正式批准设立厦门自贸试验区，面积43.78平方公里，赋予投资自由化、贸易便利化、金融国际化等先行先试权限。以此为契机，逐步推行关检“一次申报、一次查验、一次放行”通关模式，加快构建国际贸易“单一窗口”。在机场和五通码头建成10条自助边检通道。成为全国首批入境粮食指定口岸。扩大出口信保扶持覆盖面，受惠企业近2000家，增长一倍。完成出口531亿美元，占全省出口额近一半，外贸竞争力居全国第五位。

　　对外影响力进一步扩大。第18届投洽会聚焦“一带一路”战略，构建中国-中东欧国家投资合作平台，国际投资风向标作用更加凸显。亚太经合组织（APEC）海洋部长会议首次在厦举办，通过《厦门宣言》。厦门国际海洋周吸引了40多个国家和国际组织的积极参与。中美省州旅游局长合作发展对话会议提升了厦门旅游国际知名度。全球孔子学院大会在厦成功举办，来自世界1000多位知名大学校长、院长参加。出席首届中国-新西兰市长论坛，深化与惠灵顿等友城合作，与法国尼斯结好，连续四次荣获国际友城交流合作奖。隆重纪念陈嘉庚先生诞辰140周年，习近平总书记致信集美校友总会，号召广大华侨华人弘扬嘉庚精神，共圆民族复兴之梦。

　　（四）城乡统筹实现新跨越

　　高标准建设基础设施。地铁1号线全面施工，2号线跨海段正式动工。岛内火车站改造进展顺利，春运期间可投入使用。翔安机场选址获批，完成6平方公里填海造地。高崎机场T4航站楼建成启用。国际领先、国内首个远海自动化集装箱码头试运行，在我国港口发展史上写下一页。厦成高速建成通车。长泰枋洋水利枢纽工程大坝动工，库区移民推动有力，引水隧洞工程量完成84%。电力进岛第四通道投入运营。开工建设柔性直流科技示范工程，解决了不改造线路即可增加供电量的技术难题。西部垃圾焚烧发电厂、集美污水处理厂扩建工程投用，澳头污水处理厂基本建成。

　　高要求实施城市管理。抓住岛内火车站改扩建、列车不进岛的时机，实施岛内11项跨铁路畅通工程。对BRT和公交优化线路、增加班次，保障了每天10多万乘客出行。为提高效率、方便旅客，将厦金“小三通”整合至五通码头，逐班通关改为连续通关，实现航程缩短、便捷有序。为全面提升鼓浪屿旅游环境和品质，将厦鼓旅游客运航线转至东渡和嵩屿，合理分流游客和居民，实现客轮升级，缓解鹭江道交通拥堵状况。实行大城管模式，加大占道经营整治和交通秩序整顿力度，拆除违建447万平方米，城市综合管理取得成效。

　　高水平抓好“三农”工作。尽管农业比重不高，但事关全市30多万农民生产生活，我们始终高度重视，大力促进农业增效、农村发展、农民增收。建设4000亩高标准基本农田、6800亩现代设施农业、14个“菜篮子”基地。成立11家远洋渔业企业，首批31艘远洋渔船投用。扶持农产品深加工，农业产业化龙头企业产值367亿元。美丽宜居乡村建设顺利推进，建成村道49公里，房前屋后植树绿化9万平方米。转移农村富余劳动力2.7万人。多渠道开辟收入来源，农民财产性收入逐步增长，生活水平不断提高。

　　高起点建设生态文明。划定生态控制线981平方公里。岛外四区一次性通过国家生态区验收。实行“河长制”，着手整治岛外9条溪流污染源，46个村庄实施污水就地处理。岛内入海排污口截流改造基本完成。海域清淤2068万立方米。植树造林1.3万亩，封山育林15万亩，治理水土流失7500亩。严管建筑废土砂石，整治渣土车，淘汰黄标车1.7万辆。倡导低碳出行，建成公共自行车道136公里。道路清洗面积增加100万平方米。空气质量优良率达95.3%。

　　（五）对台交流合作取得新进展

　　经贸合作富有成效。新批台资项目167个（含第三地），增长21%；合同利用台资4.4亿美元，增长1.7%。对台进口54.9亿美元，出口15.6亿美元。台湾水果进口量保持大陆首位。两岸冷链物流试点项目、台湾水产公共保税分拨中心投用。两岸海运快件常态化运营。海峡两岸间集装箱运价指数正式发布。两岸贸易中心一期全部入驻，二期开工建设。两岸区域性金融服务中心核心区建设加快推进。中国银行厦门市分行获准向台湾调运人民币现钞。全省首家两岸合资基金公司投入运营。台湾半数以上银行总行委托厦门16家银行办理人民币清算，结算金额增长130%。

　　交流往来日趋活跃。第六届海峡论坛举办18项配套活动，又发布8项促进闽台交流合作政策，参会台胞达1.2万名。全国台联两岸社区交流基地落户厦门。台湾社团累计在厦设立代表机构20个。聘任台胞参与社区工作，500名社会工作者赴台培训。率先实施台湾特聘专家制度。经厦赴台旅游人次增长一倍以上。顺利举办台交会、文博会、两岸乐活节等重要活动40多场。两岸民间艺术节首次入台展演。各行业多领域交流活动频繁，厦门成为大陆与台湾交流往来最密切的城市。

　　（六）民生福祉得到新提高

　　社会事业全面进步。全市民生支出占一般公共预算支出的七成以上。完成年度为民办实事项目。建成中小学项目17个，开建公办幼儿园20所，分别新增学位2.3万个和5000个。农村义务教育寄宿生补助由每生每天8元提高到10元，非寄宿生营养膳食补助由每生每天5元提高到6元。新增病床位2000张。第一医院内科综合楼、弘爱医院、龙邦妇产医院等动工建设，市儿童医院投入使用。新建、改造标准化村卫生所52个，首批50名定向培养的村医驻村执业。故宫博物院外国文物馆落户鼓浪屿。在仁川亚运会获4枚金牌，在省运会获总奖牌数第一。2015厦门国际马拉松打破男女赛会纪录，男子冠军创国内最好成绩。民族宗教、人防、海防、科普、气象、防震、档案、地方志、妇女儿童、青年、老龄、残疾人、慈善等工作都取得新成效。

　　就业社保同步推进。全年新增就业18.3万人。实行小企业贷款贴息与员工稳定就业挂钩，有效降低失业率。城乡居民基本医疗保险财政补助标准由390元提高到430元。实现本市参保人员省内异地就医即时结算，试行省外就医就地一站式结算，有效缓解患者资金压力大、报销周期长、往返次数多等问题。实施补充工伤保险，建立多层次工伤保险体系。提高企业退休人员基本养老金，月人均达2838元。开展养老服务业综合改革试点，11家养老服务机构纳入医保范围。统一城乡低保标准，每人每月提高到550元。对低保户、残疾人、重点优抚对象等给予住房修缮补助，改善了特困群体的住房条件。建成保障性住房1.3万多套，开建5000多套，选房分配1.2万多套。

　　社会治理成效显著。以美丽厦门共同缔造促进社会治理创新，403个村居建成网格化服务管理信息平台，获评全国和谐社区建设示范城市。扶持18家民办社工机构，专业服务老年人、残疾人、社区矫正人员等特殊群体。全面开展隐患排查治理，启动新一轮企业安全生产标准化建设。全国治理餐桌污染现场会在厦召开并总结推广经验。平安厦门建设深入推进，大案要案侦破率100%，上访批次下降12.7%。军民融合深度发展，拥军优属成效显著。第四届全国文明城市顺利通过复查。

　　（七）政府自身建设取得新成效

　　深入开展党的群众路线教育实践活动，严格落实中央八项规定精神，开展楼堂馆所、会员卡、会所专项清理，加强勤政廉政教育，严格执行廉政准则，强化行政监察和审计监督，政风建设取得明显成效。全市公务接待、公务用车、公务出国（境）经费支出减少30.3%。市级考核检查、评比表彰和认定项目减少86%，文件、会议各减少20%。主动公开政府信息2.5万多件。加强依法行政，规范自由裁量权。提请市人大常委会审议法规草案4件，制定修订政府规章、规范性文件51件。获评中国法治政府第四名。自觉接受人大法律监督、工作监督和政协民主监督，认真办理市人大代表建议300件、市政协提案545件。

　　各位代表！过去的一年，我们在克服困难中前行，在抓住机遇中提升，在谋划未来中转型，做到稳中有进、稳中有为，成绩来之不易。这是在市委坚强领导下，在人大代表、政协委员有效监督和大力支持下，全市人民齐心协力、艰苦奋斗的结果。在此，我谨代表市人民政府，向全市人民，向全体人大代表和政协委员、各民主党派、工商联、各人民团体、无党派人士、离退休老同志和社会各界人士，向中央和省驻厦单位、驻厦部队、武警官兵、公安干警，向来厦的投资者和劳动者，向关心支持厦门发展的港澳同胞、台湾同胞、海外侨胞和国际友人，表示衷心的感谢和崇高的敬意！

　　我们也清醒认识到，当前发展还存在不少困难和问题:一是经济下行压力依然较大，消费需求不强，出口增长乏力；二是海域、河道污染严重，水环境治理任务艰巨；三是燃气安全监管还存在薄弱环节，城市公共安全保障能力有待提高；四是全市汽车保有量四年间从40万辆增至83万辆，交通拥堵日甚；五是政府职能转变还不到位，干部创新意识、担当意识、服务意识有待增强。我们要正视这些问题，认真加以解决。

　　二、2015年工作建议

　　今年是全面深化改革的关键之年，是建设自贸试验区的开局之年。我们要牢牢把握中央支持福建加快发展的重大历史机遇，认识新常态、适应新常态、引领新常态，发挥好经济特区服务全国全省大局的排头兵作用，努力推进科学发展跨越发展。

　　政府工作总体要求是:全面贯彻党的十八大和十八届三中、四中全会精神，深入贯彻落实习近平总书记系列重要讲话和对福建工作的重要指示精神，坚持稳中求进工作总基调，以提高经济发展质量效益为中心，深入实施美丽厦门战略规划，大力推进产业转型、城市转型、社会转型，狠抓改革开放，突出创新驱动，加强民生保障，促进经济持续健康发展和社会和谐稳定，为建成美丽中国典范城市打好基础。

　　经济社会发展主要预期目标为:地区生产总值增长10.5%左右，全社会固定资产投资增长19%以上，外贸出口增长6%，实际利用外资增长6%，财政总收入和地方级财政收入均增长10.5%，社会消费品零售总额增长13%，城镇居民和农民人均可支配收入分别增长9.5%和10.5%，居民消费价格涨幅控制在3%左右，完成国家和省下达的节能减排任务。

　　重点做好八个方面工作:

　　（一）持续构建现代产业体系，推动经济平稳健康发展

　　围绕抓龙头、铸链条、建集群，聚焦重大产业项目，着力提质增效升级，在优化结构中稳增长，在创新驱动中促转型。

　　壮大先进制造业实力。加快软件园三期、清华紫光集成电路产业园等建设，促进产业集群化、规模化、高端化。加快联电、电气硝子、ABB工业中心、天马微电子二期等龙头项目建设，带动产业链形成。扶持成长型中小企业发展，培育壮大一批本土骨干企业。加快企业技改步伐，促进传统产业转型升级。实施“机器换工”计划，提升企业生产自动化、智能化水平。深入实施质量强市战略，推进品牌带动和标准化建设。

　　强化创新驱动导向。围绕建成国家创新型城市，健全以企业为主体的创新体系，加快科技孵化器、中试基地建设，强化产学研结合，加速科技成果转化。做强南方海洋研究中心，建设清华海峡研究院、北大创新研究院、石墨烯工业技术研究院等重大平台，发展生物与新医药、新材料等新兴产业。继续创建国家信息消费示范城市、中国软件名城，完善三网融合发展体系和信息基础设施，加快发展云计算、大数据、北斗卫星应用等产业集群，推进信息消费和数字产业园建设。完善人才政策体系，深入实施“海纳百川”人才计划。

　　大力发展现代服务业。壮大航运物流业，鼓励发展国际中转业务和第三方物流，开辟更多国际航线，扩大货源腹地，加快陆地港建设，拓展海铁联运新通道。扶持发展金融业，加快建设两岸区域性金融服务中心，落实好加快金融业和现代保险服务业发展政策，支持申办民营银行，鼓励发展互联网金融，防控金融风险。做大会展业，加快建设会展四期，引进成规模的国内外展会，培育服装时尚、健康产业等专业展会，提升投洽会国际品牌影响力。做强旅游业，充分挖掘资源，完善配套设施，发展邮轮、游艇、帆船经济，提高游客满意度。加快发展研发设计、服务外包、检验检测认证等生产性服务业。鼓励企业来厦设立经济总部。

　　加快发展特色现代农业。继续建设高标准农田和现代农业设施，加快建设同安国家农业科技园、省级农民创业基地，加强渔港建设，培育推广家庭农场、农业合作联社、公司＋农户等新型农业经营模式。重点发展远洋捕捞、高科技种苗、生态高效种养、农产品加工和休闲观光农业。优化“菜篮子”产品结构，强化农产品质量监管。扶持农业产业化龙头企业，提升农产品深加工水平，打造一批优质品牌，不断促进农业增效、农民增收。

　　（二）持续深化改革，增强特区发展活力

　　紧紧围绕发展面临的突出问题，大胆探索、勇于攻坚，力争重点领域和关键环节改革取得新突破。

　　加快政府职能转变。继续理顺市、区机构职能，明确工作职责。稳步推进事业单位分类改革。加大政府购买公共服务力度。全面推进“多规合一”，坚持一张图纸管控到底、一个平台协同管理、一张表格受理审批、一套机制保障运行。加快审批流程再造，继续扩大网上审批覆盖面，减少部门、精简环节、节省时间。

　　深化经济领域改革。深入推进综合配套改革，用好先行先试政策。强化商事登记后续监管，完善商事主体信用体系。支持民营经济发展，拓宽民间投资领域。大力发展混合经济。稳步推进预算公开，建立跨年度预算平衡机制，加强政府性债务管理。整合财政专项资金，创新投融资机制，设立城市发展基金和产业引导基金。

　　推进资源要素市场化配置。改革不动产统一登记制度，加快农村土地承包经营权、集体建设用地使用权、农村房屋所有权等确权登记颁证。加快资源型产品价格改革，合理调整水资源费征收标准。

　　（三）持续扩大开放，提升国际化水平

　　发挥经济特区的开放领先优势，抓住机遇、主动作为，在构建开放型经济新体系中实现更有效率、更可持续的发展。

　　全力推进自贸试验区建设。紧紧抓住制度创新这一核心，形成与国际投资贸易通行规则相衔接的基本制度框架。推动投资便利化，实行准入前国民待遇和负面清单管理模式，将清单以外领域的外商投资项目核准制、外商投资企业合同章程审批制全部改为备案制，实施境外投资备案管理。推动贸易便利化，创新贸易监管制度，建立货物状态分类监管模式，促进内外贸一体化。推动金融业开放创新，落实人民币跨境使用、人民币资本项目可兑换、利率市场化和外汇管理等改革试点，促进实体经济发展。推动服务业扩大开放，落实跨境电商、银行服务、融资租赁、增值电信等领域开放措施，促进自贸试验区和“一区三中心”联动发展。

　　提升开放型经济水平。主动融入国家“一带一路”战略。继续实施外贸稳增长政策措施，推动外贸转型升级。建立全市统筹的招商机制，大力拓展“三维”招商，瞄准世界500强、领军台企、大型央企和优质民企，引进一批龙头项目。加快厦漳泉同城化步伐，扎实推进省内协作经济区建设，深化与闽粤赣十三市对接合作，促进区域经济协同发展。

　　营造城市国际化环境。立足举办高层次重大国际会议，着力改善硬件设施、提高服务水平，提升国际知名度和美誉度，积极打造世界性的会议中心城市。广泛开展友城合作，建立常态化交流机制。继续办好南洋文化节，争取设立中国-东盟海洋合作中心。推动公共设施与标识体系国际化。实施外国人72小时过境免签政策。增设自助边检通道，实现人员出入境更加便利。

　　（四）持续实施跨岛发展战略，推进岛内外一体化

　　围绕全域城市化，提升城乡规划、建设与管理水平，构筑协调发展的城乡区域格局。

　　优化城市空间格局。岛外要强化新城产业支撑，加快完善公共建筑和服务配套，尽快形成人气商气；继续做好旧村改造、新村建设和老区山区提升工作，加快美丽宜居乡村建设。岛内要严控建设规模，降低开发强度和建设密度，加快产业“退二进三”,进一步提升功能品位，做好鼓浪屿申遗和整治工作,打造高档商圈、旅游会展、总部经济等高端服务业集聚区。

　　加快重大基础设施建设。加快建设地铁1号线，全面建设2号线，开工建设3号线，确保工程质量、安全和进度。完成翔安机场各项目各环节的审批，抓紧机场总体规划、主体建筑、集疏运体系等设计，全面推进填海造地、征地拆迁和配套设施建设。加快东南国际航运中心、国际邮轮母港、码头自动化建设。启动吉永泉铁路厦门支线前期工作，抓紧建设动车运用所、前场铁路大型货场。推进海沧隧道、同集路改造等“两环八射”城市快速路网建设。构建公共自行车道系统。全力以赴推进长泰枋洋水利枢纽工程,尽快建成让全市人民饮用更好水质的第二水源。建成莲花水库。加强电网建设，提高用电可靠性和稳定性。

　　推动城市管理精细化。提高数字城管水平，构建市、区、街（镇）、社区四级城市综合管理网络。继续整治“两违”，严管占道经营、占道停车、施工扬尘、运输车辆跑冒滴漏、油烟噪声扰民等，有序推进垃圾分类工作。推动地下管网综合管廊建设，及时处理道路破损、井盖缺失、路灯故障等问题。

　　（五）持续深化两岸融合，更好服务大局促进发展

　　发挥前沿平台优势，用好用活用足对台先行先试政策，推动交流合作向更宽领域拓展。

　　加快产业对接。实质性推进两岸新兴产业和现代服务业合作示范区建设，为项目进驻创造条件。增创台商投资区新优势，在服务好现有台资企业、鼓励其增资扩产的同时，着力引进台湾产业龙头项目，更加重视创新型中小企业招商，加大台湾人才引进和创业扶持，促进与台湾企业优势互补，共铸链条、共建集群、共同发展。

　　推动往来便利。依托“小三通”、“大三通”，进一步创新口岸管理服务体制机制，促进直接往来便利化，在海峡旅游、海运快件、车辆互通等方面先行先试，提升两岸人员、货物进出厦门口岸效率，让两岸往来更快捷顺畅。

　　增进情感融合。发挥海峡同根同源、同文同宗、两岸一家亲优势，办好第七届海峡论坛，扩大台交会等品牌影响力，密切科技、教育、文化、医疗等领域合作，加强基层、民间和青少年交流，增进两岸民族文化认同。维护在厦台胞合法权益，鼓励建言献策、参与社区管理，让台胞在厦工作生活更加温馨舒适。

　　（六）持续完善公共服务体系，全面繁荣社会事业

　　坚持建机制、补短板、兜底线，着力解决群众最关心最急迫的问题，让发展成果惠及全市人民。

　　推动基本公共服务均等化。立足促进社会公正、增进人民福祉，让全体市民享有均等的教育、卫生、养老等基本公共服务。通过加大财政统筹和转移支付力度，扩大有效供给，培育岛外优质资源，推动公共服务在城乡、区域、群体间均衡发展。在新城区建设中充分预留社会事业发展用地，商业和综合服务设施占新建社区总建筑面积不低于10%。政府公房优先用于公共服务需要。加快构建公共服务绩效评价体系，着力提高服务质量和人民群众满意度。

　　促进各类教育协调发展。新建19所公办幼儿园，新改扩建21所中小学，分别增加学位5000个和2万个。全面推进素质教育。高标准完成国家义务教育均衡发展试点任务。完善现代职业教育体系，壮大我市发展急需的技工队伍。推动高校内涵发展，提升协同创新能力。支持和规范民办教育，鼓励中外合作办学。深化校企合作，促进产教融合。

　　提升市民健康水平。深化医药卫生体制改革，完善分级诊疗制度，通过提高医保支付比例，引导更多患者到基层医疗机构就诊。推进药品集中采购和统一配送。加快已批医院建设，鼓励社会资本发展高端医疗、专科医疗等健康产业，满足多元医疗服务需求。完善公共卫生体系，加强重大疫情防控，提高应急救治水平。加强医德医风建设，构建和谐医患关系。坚持计划生育基本国策，提高出生人口素质。完善体育设施建设，推动群众体育和竞技体育全面发展，广泛开展全民健身运动。

　　增强文化软实力。践行社会主义核心价值观，全面提升社会公德、职业道德、家庭美德和个人品德。加快各类文化设施建设，加强文物保护。加大文化产业扶持力度，办好文博会等重大文化活动，策划打造一批文艺精品。

　　（七）持续改善民生，加快和谐社会建设

　　尊重人民群众主体地位，鼓励和支持社会各方参与，创新治理方式，维护公平正义，保障市民安居乐业。

　　加强社会保障。完善就业创业扶持体系，开展新型职业农民教育培训，做好高校毕业生、退役军人等就业服务。推进企业工资集体协商，逐步提高最低工资标准，落实职工带薪休假制度，深入创建和谐劳动关系。继续提高低保标准，加大救助力度，健全城乡保障体系。完善参保人员自付医疗费困难补助政策。鼓励各类资本建设老年养护设施。关爱残疾人，发展慈善事业。新开工保障性住房5000套。

　　推进社会治理。深化美丽厦门共同缔造，坚持“核心在共同、基础在社区”，积极探索共谋共建共管共评共享的城乡治理体制机制，发挥全国社区治理和服务创新实验区示范作用。组织好村居换届选举，提升基层组织自治能力。扎实推进全国文明城市新一轮创建。促进诚信建设和志愿服务工作制度化，做好关心下一代工作，加强未成年人思想道德建设。全面推进平安厦门建设，创新立体化社会治安防控体系，完善多元化纠纷解决机制。化解信访积案，提高办结率。加强食品药品市场监管，强化生产安全，优化应急联动和防灾减灾救灾机制，建设突发公共事件预警信息发布平台，创建全国安全发展示范城市。继承发扬双拥优良传统，力争全国双拥模范城市“九连冠”。

　　（八）持续加强生态文明建设，提升城市宜居度和竞争力

　　推动绿色循环低碳发展，继续开展宜居环境建设，打造青山绿水、碧海蓝天的美丽家园。

　　优化生态环境。加快建设国家海洋生态文明示范市，扎实推进海洋生态治理和岸线保护。以水清无味为目标，重点整治9条溪流，继续推进九龙江流域治理，加大污水截流处理力度，推进岛外重点片区污水管网和污水处理厂建设。不断推进汽车烧油改烧气、道路清扫改清洗、绿化养护冲灌改喷雾，增加绿地、降低扬尘、清新空气。争创国家生态市、生态园林城市。

　　推进资源节约。坚持土地开发与项目投资强度、建设进度、投入产出等紧密挂钩，提高节约集约利用水平。加快淘汰落后产能，支持节能低碳产业和新能源、可再生能源发展。提倡低碳生活，节约能源资源，大力发展循环经济，建设资源节约型、环境友好型社会。

　　完善生态文明制度。实行生态文明建设和环境保护目标责任制，建立资源环境承载能力监测预警机制，健全生态文明考核评价体系，制定生态红线管理办法和生态补偿转移支付办法。推行环境污染第三方治理。完善环境监测网络，严肃生态环保责任追究，严厉打击环境违法行为。

　　三、以法治思维和法治方式，加强政府自身建设

　　法治是现代城市治理的基石。我们要运用法治思维和法治方式深化改革、推动发展、化解矛盾，持之以恒建设法治政府，切实加快城市治理体系和治理能力现代化。

　　坚持依法行政。将依法行政贯穿政府工作全过程，遵循法无授权不可为、法定职责必须为，全面推行权力清单制度，做到行政权力进清单、清单之外无权力。进一步完善政府规章和规范性文件、重大决策合法性审查机制，做到重大改革于法有据、决策立法紧密衔接。推进综合执法，加强基层力量，做到规范公正文明执法。开展“六五”普法，在全社会弘扬宪法精神，努力构建办事依法、遇事找法、解决问题用法、化解矛盾靠法的法治社会。

　　坚持科学履政。汇聚各方智慧，用好第三次全国经济普查成果，科学编制“十三五”规划。重大决策坚持公众参与和专家咨询，加强社会稳定和行政决策风险评估，落实全程跟踪反馈和终身责任追究。加强政务平台建设，完善市长专线、政府新闻发言人制度。自觉接受人大及其常委会法律监督、工作监督和政协民主监督，认真办理人大代表建议和政协提案，广泛听取民主党派、工商联、无党派人士和工会、共青团、妇联等人民团体意见。

　　坚持从严治政。以务实作风改进政风，巩固和拓展党的群众路线教育实践活动成果，深化“四风”整治，践行“三严三实”，推动作风建设常态化。严格执行中央八项规定精神和国务院“约法三章”，严控各类评比表彰、节庆论坛，规范“三公”经费等一般性支出，强化公务卡结算管理，切实把艰苦奋斗、厉行节约体现在政府运行各环节。以严明纪律畅通政令，把抓落实作为提升政府公信力的着力点，作为检验干部执行力的试金石。继续治理庸懒散奢，整治行政不作为、乱作为、慢作为等问题。对确定的目标、制定的政策、部署的任务，必须一抓到底，做到事有专管之人、人有明确之责、责有限定之期。

　　坚持廉洁从政。始终保持反腐败高压态势，对腐败问题“零容忍”。强化行政监察和审计监督，完善政府内部层级监督和专门监督。切实加强反腐倡廉制度建设，对权力集中的部门和岗位实行分事行权、分岗设权、分级授权，强化内部流程控制，防止权力滥用。积极推进预防职务违纪和职务犯罪制度建设。全面推进政务公开，实现决策、执行、结果公开透明，让监督的阳光照射到权力运行的每个角落。

　　各位代表！厦门发展进入新常态，迎来新机遇。让我们紧密团结在以习近平同志为总书记的党中央周围，在市委的领导下，同心协力，开拓创新，为建设美丽中国典范城市而努力奋斗！
